# Remote Control of Cardiac Implantable Electronic Devices: Exploring the New Frontier—First Clinical Application of Real-time Remote-control Management of Cardiac Devices Before and After Magnetic Resonance Imaging

**DOI:** 10.19102/icrm.2019.100102

**Published:** 2019-01-15

**Authors:** Esteban M. Kloosterman, Murray Rosenbaum, Brian La Starza, Jamil Wilcox, Jonathan Rosman

**Affiliations:** ^1^Boca Raton Regional Hospital, Boca Raton, FL, USA; ^2^Charles E. Schmidt College of Medicine, Florida Atlantic University, Boca Raton, FL, USA

**Keywords:** CIED programming, MRI-safe mode algorithm, remote control, remote monitoring

## Abstract

The purpose of the present study was to evaluate the performance of remote-control (RC) management of cardiac implantable electronic devices (CIEDs) in clinical practice using a new service model in patients undergoing magnetic resonance imaging (MRI) scans. The number of CIEDs is constantly growing, alongside the demands for prompt checks. Although remote CIED interrogation exists, ultimately, real-time remote management is the goal. In this study, patients with MRI-conditional devices suitable for RC interaction who required an MRI were enrolled. An onsite technician began the RC session by contacting the remote operator, applying the programmer wand, and keying in an access code. The device was remotely checked via encrypted Wi-Fi by an electrophysiologist using a laptop. An MRI-safe mode was programmed per a preestablished proprietary algorithm. Following the scan, patient devices were remotely reinterrogated and reprogrammed to baseline, with adjustments made as clinically necessary. Patients subsequently were asked to complete a survey. Ultimately, a total of 100 RC CIED reprogrammings were performed in 50 MRI sessions, prescan and postscan. The average RC time interaction was four minutes prescan and three minutes postscan, respectively. No complications occurred. Five patients had more than one MRI in this study and 15 patients had had previous MRIs. In eight patients, baseline settings were reprogrammed. Most patients (82%) were very satisfied, preferring device specialist remote management. Only 14 (32%) patients used home remote monitoring. In conclusion, RC management of CIEDs in the MRI setting is feasible, safe, and clinically relevant. Use of the MRI mode determination algorithm was safe, consistent, and efficient. Expanding RC in CIED management for service anytime, anywhere is the next challenge.

## Introduction

For the past 10 years, our motto has been: *If a robot on Mars can be remotely controlled from Earth, we can likely remote control (RC) a pacemaker in Boca Raton, Florida*.^1,2^

The use of cardiac implantable electronic devices (CIEDs) has evolved exponentially over the last decade, leading to the need for a prompt response at the time of device interrogation and check. Significant advancement has been made in the use of home remote monitoring and, to some degree, hospital remote “checkups,” which has substantially decreased the number of office visits and bedside manpower required for standard cardiac device follow-up and management. However, as of today, the only system available to reprogram a CIED of any kind requires “bedside” implementation, ie, the physical presence of specialized personnel (eg, doctor, physician’s assistant/nurse practitioner, company representative) to operate a dedicated device programmer.

The development of remote interaction with cardiac devices should come as no surprise when we consider that these are electronic devices with the capability to connect and communicate.^3–5^ We began to explore remote real-time CIED management with online programmer mirror screen visualization and “guided reprogramming” using a laptop attached to a programmer and a remote iPad (Apple, Cupertino, CA, USA).^6,7^ The system was tested in an emergency room setting with an impressive average of a 2.3-minute response time, in comparison with a 69-minute response time for a company representative service.^8^ Subsequently, we gained experience introducing the concept of express check via a dedicated server with assisted-reprogramming when needed; in this case, the interaction was carried out via direct communication with a programmer using an encrypted Wi-Fi connection. This model allowed for remote movement of the programmer cursor, but without functionality capabilities: instead, the bedside assistant still had to touch the screen. This system was tested in cardiologists’ offices and resulted in a successful option of prompt, readily available CIED checks any day, any office time. This is a significant departure from the conventional device “clinic,” which is in many instances dependent on the services of company representatives.^9^

Once the ability to remotely interrogate and reprogram CIEDs became a technological reality, it required a clinical setting to be tested in. Today, all magnetic resonance imaging (MRI)-conditional CIED devices require a change in their pacing mode to an “MRI-safe” mode to block or avoid the effects caused by the electromagnetic interference generated by the MRI scanner. Thus, the MRI setting represents an ideal opportunity to test the concept of RC, given the inherent need for device reprogramming pre- and post-MRI study. The conventional service model usually requires the physical presence of a device company’s representative (or otherwise proficient medical personnel) to handle the programmer at the patient’s side.

As a side note, there are currently no definitive guidelines in choosing an MRI-safe mode. A physician’s order is required for the determination of the mode that the device ought to be programmed to in order for the patient to correctly undergo the procedure. The reality of today’s system is that there are several deficiencies at many levels, including the scheduling of this type of patient, the readiness of physicians’ orders, and the availability of the programmer and company representative both prestudy and poststudy. Usually, overextending the representative role covers for clinical gaps in the process. Other than the mode change, there is no adequate systematic interpretation of the rest of the device interrogation findings that are exposed during these interactions. This type of service model has been deemed unsustainable in the near future of health care for many reasons, including increasing numbers of MRI-conditional devices and the fact that MRI scan studies must be performed with a significant requirement of human resources in the field to cover these and other services, of which many companies are in short supply of. Additionally, there are several operative inefficiencies that can be improved for better patient care, compliance, and institutional flow.

**Figure 1:**
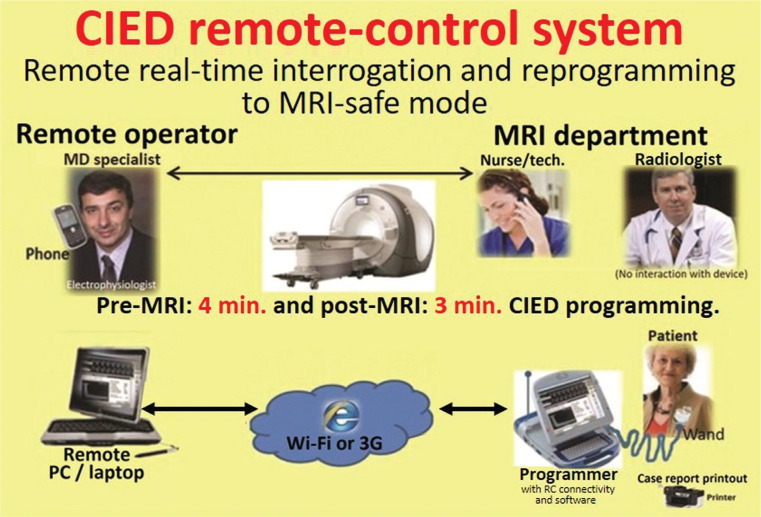
Workflow layout of the present study evaluating RC management of cardiac devices pre- and post-MRI.

We tested an alternative service model using RC management of CIEDs that would allow for the expeditious and safe device analysis and programming of MRI-conditional cardiac devices without the need for a proficient onsite operator or office device preevaluation **(Figure 1)**.

**Figure 2:**
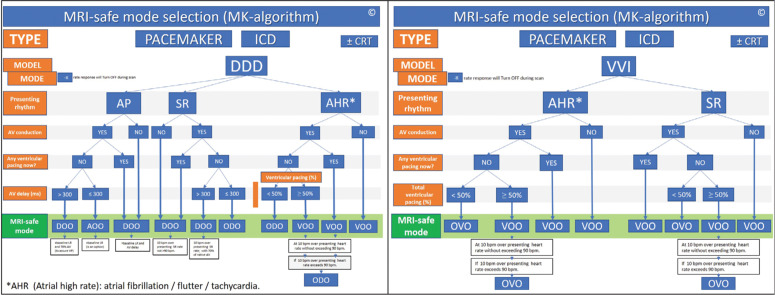
The algorithm for the selection of the MRI-safe scan mode in the case of dual-chamber DDD or single-chamber VVI modes.

RC of CIEDs in the MRI setting may also allow for more flexible scheduling of patients without compromising the usual technical qualified support that the MRI centers receive as a result of the onsite presence of company representatives and could actually maintain or improve the quality of the check with expert input. Additionally, in the RC model, MRI-safe mode selection is performed using a proprietary algorithm aimed at standardizing the decision-making process **(Figure 2)**.^10^

In this article, we present what is believed to be the first reported experience of RC management of CIEDs. The aims of this study were (1) to evaluate the use of real-time RC—including interrogation and reprogramming at the MRI site, feasibility, safety, and clinical relevance—and to compare the quality of such to the present service model involving the physical presence of a company representative; and (2) to evaluate the performance of an algorithm for decision-making around the MRI-safe mode for the purpose of having the devices programmed pre-MRI.

## Materials and methods

This was a single-center, nonrandomized study that employed a 1.5-T MRI scanner. The study target was 50 MRI scan sessions with pre- and post-CIED RC management. Consecutive patients attending the Boca Raton Regional Hospital in Boca Raton, Florida for an MRI scan who had an implanted pacemaker or defibrillator MRI-conditional system (Medtronic, Minneapolis, MN, USA) were invited to participate in the study.

To ensure its compliance with current rules and regulations, the study protocol was presented for review and approval by an external institutional review board committee. A specific institutional review board- and hospital-approved informed consent protocol was utilized for patient enrollment in the present study.

Prior to enrollment, patients underwent a routine MRI prescreening evaluation to confirm the following inclusion criteria: (1) the patient was at least six weeks postimplant and (2) they had a pectoral implant and no lead extenders, adapters, or abandoned leads.

Patients with either non-Medtronic-branded or Medtronic-branded, non-MRI-conditional systems were excluded from this study.

### Cardiac implantable electronic device management system, study, and communications protocol

For this study, a 2090 Medtronic (Minneapolis, MN, USA) programmer was used with RC software installed for the 2090 Programmer, Analyzer Software, 90003/S326 (Food and Drug Administration approval gained October 19, 2015). In addition, the programmer had a connectivity modem card to allow for Internet connection. Internet access was obtained via one of two modalities, either a direct Ethernet cable connection or a wireless modem to a dedicated cellular “hotspot.” The remote Wi-Fi connectivity for the remote laptop included an Ethernet cable, local Wi-Fi, cellular connection, and a modem for international mobile transmissions (Skyroam™; Skyroam Ltd., Shenzhen, China).

The “programmer” was a dedicated device company computer used to communicate with the patient’s implanted device. A wand was placed over the device surface, and, after an initial “handshake,” communication was established and continued via the wand connection (or in some devices, wirelessly). The programmer allows for (1) interrogation of the device’s stored information and evaluation of the functioning of the device and (2) programming of different device features.

Following a standard of care, the patients were specifically tested for (1) pacer lead impedance between 200 ohms (Ω) and 1,500 Ω; (2) implantable cardioverter- defibrillator (ICD) lead impedance between 200 Ω and 3,000 Ω and high-voltage coils between 20 Ω and 200 Ω; (3) no diaphragmatic stimulation at a pacing output of 5.0 V and at a pulse width of 1.0 ms in patients who would be programmed to an asynchronous mode during scanning; and (4) pacing thresholds of no more than 2.0 V at a pulse of 0.4 ms.

The study protocol workflow **(Figure 1)** involved a specialized remote-operator electrophysiologist and a nurse/technician at the MRI center. The latter individual was trained in the tasks of turning on the programmer, applying the wand to the patient’s device, and keying in an access code when provided.

A radiologist was on the premises for general supervision of operations, but this person had no direct interaction with the patients or devices. A company representative was also available on the premises for redundancy and backup; again, this person had no interaction with either the patients or devices.

**Figure 3:**
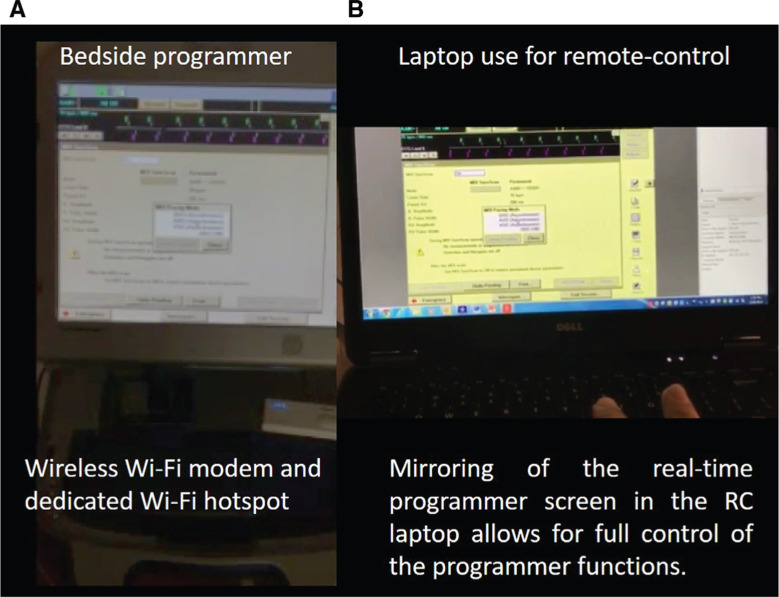
“Programmer” for the remote management of MRI-conditional cardiac devices. **A:** This computer had a connectivity modem card with options of intranet cable connection or a dedicated Wi-Fi cellular hotspot. The session was initiated by a bedside technician. **B:** Once informed that the patient was ready, the remote operator logged onto the session to access the RC functions of the programmer.

The onsite technician, after obtaining written informed consent from the patient, began the RC session by contacting/calling the remote operator. He was also in charge of turning on the programmer and applying the programmer wand to the patient’s device **(Figure 3A)**. The remote operator, once informed that the patient was ready, obtained a randomly assigned “key code” from a dedicated website (Medtronic Secured Access External Login; Medtronic, Minneapolis, MN, USA) and logged into a second site (Bomgar™; Bomgar Corp., Ridgeland, MI, USA) that allowed for remote device interaction **(Figure 3B)**. He then provided the onsite technician with the “session code” to be keyed in. The programmer subsequently automatically connected, via encrypted Wi-Fi, with the remote management website displayed on the remote operator laptop screen. Once the RC session commenced, the remote operator had complete control of the programmer functions to check and reprogram the device as needed.

The MRI-safe mode was programmed using a specific preestablished proprietary algorithm **(Figure 2)**. Postscan, patients were reinterrogated remotely and reprogrammed to baseline settings, with adjustments made as clinically necessary. A clinical report was generated after each encounter and emailed to be printed onsite, detailing the main findings and the performed interactions. The participants filled out a short customer satisfaction questionnaire at the end of the session.

### Outcome measures and statistical analysis

Outcome measures included the completion of successful communications protocols and successful RC and management of the cardiac device pre- and post-MRI scanning, with time measurements obtained for efficiency. Both the time of the RC session and the overall patient encounter with different intervals were monitored. During the RC session, laptop screenshots were captured and stored as part of the documentation process, which added some time to the session that was not discriminated in the results. The production of the final report was not part of the RC session, but was part of the overall patient encounter. In this regard, the duration of the patient’s encounter included the time required for providing informed consent and enrollment in the study.

Descriptive statistics were used to calculate means and standard deviations for continuous variables and percentages for categorical variables. Given the aim and characteristics of the study, sampling was qualitatively determined by a clinical and technical consensus.^11^ In consideration of the volume of patients with MRI-conditional devices undergoing MRI scanning that usually attend our institution as well as the time allotted and the available resources, we set the sample dimension to 50 MRI scan sessions. Fifty was established as a representative number of the phenomenal variation, providing 100 RC sessions (pre- and post-MRI scanning), which is enough to test the application, communications, and service model protocol and be noninferior to standard proceedings.

## Results

### Patients and devices

A total of 100 RC device interrogations and reprogrammings done pre- and post-MRI scan were performed in 50 1.5-T MRI scan sessions. The MRI scans performed included 12 for the brain, five for the brain/lumbar spine, 13 for the lumbar spine, one for the lumbar spine/hip, six for the cervical spine, two for the thoracic spine, two for the knee, two for the abdomen, one for the abdomen/chest, one for the shoulder, one for the wrist, one for the ankle, one for the pelvis, one for the pelvis/hip, and one for the hip. Forty-four patients were entered into the study with consecutive enrollment over a six-month period. Five (10%) of these patients underwent more than one MRI scan during the study on different dates (four patients had two MRI scans and one patient had three MRI scans).

Fifteen participants (33%) had undergone MRI scans prior to the current study. The mean age was 75 years ± 9.6 years. Eighteen participants were female (40%) and 26 were male (60%). The device distribution was 48 pacemakers (46 DDD and two VVI) and two ICDs (one DDD and one VVI).

Only two of the patients invited declined to participate in the study, and they proceeded to have their MRI scans done following the hospital conventional protocol that involved the company representative.

### Session time and feasibility

The average duration for the MRI scans was 35 minutes (range: 20–50 minutes). The calculated total average time of patients’ MRI scan service, from arrival to discharge, was 60 minutes, not including check-in at the hospital front desk. This time did include a brief interview by MRI personnel, changing to a gown for the scan, intravenous insertion if required, device check and reprogramming, scan, removal from the MRI machine, removal of the intravenous line if required, changing back into street clothes, and device check and reprogramming.

The average RC session prescan time was four minutes, whereas the average RC session postscan time was three minutes.

Remote transmission locations included the hospital premises; in town [eg, offsite hospital (n = 28), electrophysiologist’s office (n = 12)]; out of town [eg, Miami (n = 2), Orlando (n = 4)]; and international [eg, Paris (n = 1), Luxemburg (n = 3)]. There were no communication problems during the RC sessions. An occasional transmission delay, estimated to be a maximum of two seconds, was not found to be clinically significant, and so did not detract from adequate patient care. All transmission protocols were checked before enrolling each patient to ensure adequate Internet connection and speed. At no time was there a need for cancellation, but troubleshooting was performed to establish the most reliable Wi-Fi connection.

### Remote-control protocol safety and safe mode algorithm performance

No complications or adverse events occurred. There were no communication problems during the RC sessions and no need for the backup company representative to intervene during the study cases. One patient was taken to the emergency room after the MRI scan with several nonspecific symptoms that were eventually diagnosed as anxiety. The device was rechecked at bedside in the emergency room and found to have adequate function; no changes were made.

**Figure 4:**
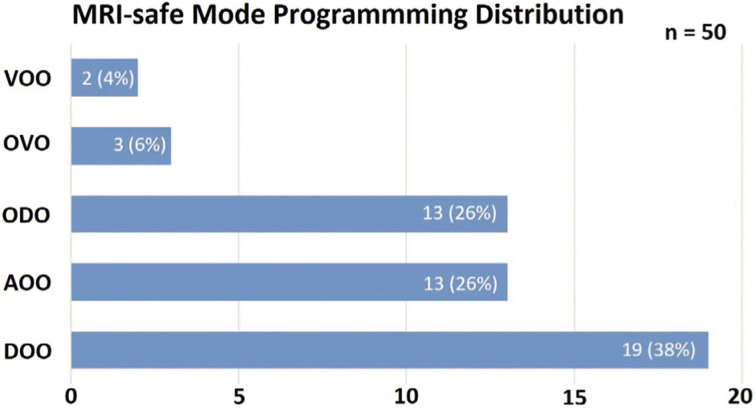
MRI-safe mode programming distribution using the provided algorithm.

The MRI-safe mode algorithm was used as conceived and was able to encompass all case presentations. As a result of a decision tree, MRI-safe mode programming **(Figure 4)** was as follows: 19 (38%) DOO, 13 (26%) ODO, 13 (26%) AOO, three (6%) OVO, and two (4%) VOO. Atrial fibrillation was found to be the baseline rhythm in four (8%) of the patients.

Owing to clinical findings and conditions in eight participants (18%), the remote operator (specialist) decided to reprogram baseline settings after the MRI scan was performed (ie, turned on rate response, changed DDDR to AAIR-DDDR, turned on atrial therapies). The patients were informed about the changes, and this was reflected in the case reports compiled for each of them.

### Patient survey

Thirty-three (75%) of the 44 patients were able to complete the postscan questionnaire; most nonresponders were limited due to an impairment of cognitive abilities. Patients’ answers are summarized in **Table 1**. Eighty-two percent (27/33) of the participants were very satisfied with their experience of having their device remotely operated. No patient reported being dissatisfied.

The majority of patients who responded to the questionnaire (25/33; 76%) felt comfortable and reported that they would have preferred to be evaluated by a specialist with RC within a regulated environment. A minority of patients (3/33; 9%) reported preferring a technician onsite for RC; the primary reason cited was the preference for more human interaction.

Of note, only 14 (32%) of 44 patients were familiar with and used home remote monitoring follow-up services for their devices (CareLink; Medtronic, Minneapolis, MN, USA).

## Discussion

To the best of our knowledge, this is the first study to investigate RC management of CIEDs. We present a new service model for CIED interrogation and reprogramming that departs from the conventional requirement of the physical of presence of a specialized bedside operator using an RC system application. We chose the MRI setting to clinically test this model given the inherent need to interrogate and reprogram the devices in a control setting.^7^

All 100 performed transmissions operated well. There were no significant delays during the sessions, despite the sometimes long distances and the variability of the remote sites (eg, hospital, in/out of town, out of the country). Prestudy, the system was tested successfully in similar settings, including in the context of international transmissions with nonimplanted devices.

Although no patient session was cancelled due to a communication problem, a precheck of the system before each interaction led to several adjustments required to be performed to assure reliable connectivity. Most variability in connectivity was caused by Wi-Fi bandwidth. Initially, a communication card in the programmer was used in connection with a wireless modem with a dedicated Wi-Fi cellular hotspot. This setup worked well multiple times, but, from the moment of initial test to the time of actual use, it was also characterized by a lack of reliability/predictability, likely due to local variations in cellular signal strength. An Ethernet cable connection to the programmer was alternatively used to promote consistent results. Such connectivity limitations were assumed to be associated with local usage variations and the phenomenon of shielding at the MRI department, as testing the communication elsewhere in the hospital setting did not reproduce these issues.

Similarly, the remote laptop Wi-Fi performance was variable depending on the location. Different connectivity modalities were used to achieve the best bandwidth and quality signal [Wi-Fi to local network, cellular subscriber identification module card integrated to the laptop, international roaming Wi-Fi (Skyroam™; Skyroam Ltd., Shenzhen, China), and Ethernet cable connection]. In an eventual established clinical setting system, the best connectivity option would be set onsite and remotely based on stable, reproducible variables to achieve the fastest, most reliable communication protocol possible. Part of the learning experience of the study was to try and troubleshoot different communication alternatives while adjusting to dynamic local needs.

We acknowledge that cybersecurity is a concern in the use of this type of model; however, this should be treated like any other situation in which communications and interactions with sensitive material occur in cyberspace (eg, airlines, banking, military). Protocols should be adjusted accordingly.

In this study, we used several layers of security, including computer access passwords, encrypted protocols, and two different servers (one to obtain an “access key code” by the remote operator, given to the onsite technician via telephone communication, and the second server to use with a separate access code to remotely control the programmer). Further standards will need to be determined to enable use on a wider scale.

The duration of RC interactions averaging four minutes and three minutes during prescan and postscan can certainly be cut down instead to three minutes and one minute, respectively. The additional time was used to generate a log of “print screens” for the study documentation purposes that could be avoided otherwise. The generation of a postevent report (including the pre- and post-MRI scan interactions) took four minutes to five minutes and was not part of the RC session. This information was emailed separately; however, a printout report from the programmer was generated remotely, in real time, at bedside.

Although most of the enrolled subjects were very satisfied, further patient education will be needed to promote trust in the new concept and system. To begin with, patients are generally unaware that device interaction and reprogramming is needed at the time of MRI scanning; during this study, many assumed that they could just “walk into” the machine. Additionally, a more expanded use of CareLink home remote monitoring (Medtronic, Minneapolis, MN, USA) would help to further emphasize that the device information can be accessed remotely as a routine event.

Radiologists would also benefit from education regarding the workflow required not only to establish that a system is MRI-conditional but also to understand the need for an order set to allow for proper testing and MRI-safe programming. The MRI-safe algorithm proved extremely useful to streamlining this process, which may eventually evolve as a standard of care order set to be used by radiologists themselves rather than by specialists. Today’s model requires in advance to “preorder” or, in some devices, “preprogram” a defined MR-safe mode, with no regard to the patient’s actual clinical circumstances at the time of the MRI scan. The presented algorithm was clinically used and tested in 55 patients before the current study, which allowed for a fast and consistent decision-making process accounting for real-time, in situ conditions. Eventually, this may lead to the automation of a CIED mode switch to MRI-safe mode in the MRI scanner environment.

The interface of this model using the present programmer will likely change, but the essence of the system concept was well-tested in this study. Conceptually, this represents a new and innovative device management and interaction option; it opens a host of possibilities beyond the specific, individual patient interchange.

The need for device reprogramming other than the MRI-safe mode was limited in this study setting, yet clinically useful when required. Settings other than the MRI wherein an RC CIED interaction could be useful are yet to be determined, along with further exploration of service models.

### Limitations

The present study was meant to be a proof-of-concept and feasibility investigation in a real clinical setting; as such, it was a single-center, single-arm, and nonrandomized but with a consecutive enrollment design investigation.

There is currently only one CIED vendor with equipment to readily perform RC (Medtronic, Minneapolis, MN, USA); therefore, the experience and results of the present study only apply to the equipment currently in existence. Yet, the general concept and service model could be applicable to other upcoming systems and interfaces.

It is important to remark that the principal author has significant experience and knows how to troubleshoot and circumvent occasionally arising technical challenges. Although the RC technology exists, it will require further adjustments and a specified protocol to be used at a large scale. In fact, one of this study’s secondary purposes was to delineate what that protocol should encompass.

The presented service model in the study was not meant to be directly compared with our standard existing service, but we have collected prospective baseline data of our regular protocol to give an adequate benchmark to what is our standard of care.

Although a full economic analysis is beyond the scope of this paper, our findings indicate that a decreasing cost of human resources can be combined with a streamlined, safe, and clinically relevant way to manage CIED in real clinical practice in patients undergoing MRI scans. In this regard, our calculated total patients’ hospital time decrease was 25 hours and there were savings of company representative working time of 80 hours to 100 hours per 50 MRI scans performed.

The cybersecurity of the information and communications protocol was intended to have several layers of protection for this study protocol. A larger-scale operation may require additional safeguards.

Although, in this case, the MRI-safe mode algorithm was only applied to the hardware of one device company, our experience with its use is applicable with all vendors.

## Conclusions

RC management of CIEDs in the MRI environment is feasible, safe, and clinically relevant, with the potential to streamline patients’ workflow. Remote communication and interaction with devices was effective, with no complications. Patients were highly receptive to this model and reported having very satisfying experiences.

The use of the MRI-safe mode programming algorithm was efficient and useful, producing concise and systematic results.

RC of CIEDs is an innovative tool that provides an answer to today’s need for expeditious service. It allows a specialist to directly connect and fully manage a CIED in a patient regardless of their respective physical locations. This kind of intervention is a significant breakthrough in our current standard of care.

The scaling and implementation of the presented RC service model to provide broad access to real-time RC CIED management anytime, anywhere is the next challenge.

## Figures and Tables

**Table 1:** Results of the Patient Postvisit Satisfaction Survey*

Question	Percentage of Patients (Number of Respondents/Total Participant Population)
Do you have CareLink at home?**	
Yes	32% (14/44)
Are you satisfied with how the device check was performed?	
Very satisfied	82% (27/33)
Somewhat satisfied	9% (3/33)
Neutral	9% (3/33)
Dissatisfied	0% (0/33)
Are you comfortable with your device having been evaluated and reprogrammed in RC fashion by an expert within a regulated environment, or you would you prefer for it to be managed in the usual way (ie, by a company technician?)	
Prefer a remotely-located expert	76% (25/33)
Do not mind one way or the other	15% (5/33)
Prefer having a company technician on-site	9% (3/33)

RC: remote control.

*Questionnaire answers were obtained from 75% of the patients (33/44 patients).

**Data for this answer were obtained from patients and a CareLink (Medtronic, Minneapolis, MN, USA) database review.
